# Sleep and Selective Memory Consolidation in Infants: Exploring the Impact of Learning Contexts

**DOI:** 10.1111/infa.70045

**Published:** 2025-09-16

**Authors:** Neele Hermesch, Sabine Seehagen, Rachel Barr, Jane S. Herbert, Carolin Konrad

**Affiliations:** ^1^ Developmental Psychology Faculty of Psychology Ruhr University Bochum Bochum Germany; ^2^ Department of Psychology Georgetown University Washington DC USA; ^3^ School of Psychology University of Wollongong Wollongong New South Wales Australia; ^4^ Faculty of Psychology Clinical Child and Adolescent Psychology Mental Health Research and Treatment Center Ruhr University Bochum Bochum Germany

**Keywords:** deferred imitation, infants, scaffolding, screen media, sleep‐dependent memory consolidation, transfer deficit

## Abstract

Post‐encoding sleep facilitates memory consolidation from early infancy. Learning from digital content might also benefit from post‐encoding sleep. However, infants find it more difficult to learn and remember screen content (transfer deficit) and may only recognize its relevance when scaffolded by caregivers. We investigated infants' memory performance as a function of presentation mode (live or video), post‐encoding sleep, and caregiver scaffolding with the aim of replicating the transfer deficit and the beneficial effect of a post‐encoding nap. We expected the nap benefit to be less pronounced when information was encoded from videos. We compared data from a live (*n* = 68) and a video experiment (*n* = 69). In both experiments, 15‐ and 24‐month‐olds watched demonstrations of two deferred imitation tasks (live or on prerecorded video). During one of the demonstrations, caregivers scaffolded the task. Half of the infants in each age‐group napped for ≥ 30 min after demonstration, whereas the others remained awake for ≥ 4 h. Memory performance was assessed after 24 h counting the reproduced target actions. Contrary to expectations, the nap benefit did not replicate in the live demonstration sample. However, when both samples were examined together there was a main effect of nap condition showing that infants who had napped retrieved more target actions than awake infants. Moreover, cross‐experiment comparisons revealed a transfer deficit and an unexpected disruptive effect of caregiver scaffolding on memory performance in 15‐month‐olds. Results are discussed in light of limits on detecting sleep‐mediated memory effects and the challenges of remembering digital information in infancy due to cognitive load.

## 1 Introduction

Sleep supports memory consolidation in adults (e.g., Diekelmann and Born 2010; Klinzing et al. 2019; Walker and Stickgold 2004). Over the past decade, there has been growing evidence that post‐encoding sleep enhances memory consolidation in infants and young children as well (Konrad and Seehagen 2020; Mason et al. 2021). Sleep‐dependent memory consolidation has been shown in infants aged between 3 and 36 months for a variety of tasks including declarative memory tasks (e.g., Friedrich et al. 2015, 2020; Horváth et al. 2018; Seehagen et al. 2019), motor learning tasks (e.g., Berger and Scher 2017), and generalization tasks (e.g., Friedrich et al. 2019; Gómez et al. 2006; Konrad et al. 2016). However, most studies were conducted with infants between the ages of 3 and 17 months of age, which has resulted in a paucity of research exploring the effect of sleep on memory consolidation in infants between the ages of 18 and 24 months (Mason et al. 2021).

Memory benefits of post‐encoding sleep in adults and children alike have been explained with the active systems consolidation theory and the synaptic homeostasis hypothesis, and both mechanisms could also work in concert to explain the effects (see Mason et al. (2021) for review). According to the synaptic homeostasis hypothesis, learning increases synaptic potentiation during wakefulness. Sleep episodes are necessary for synaptic downscaling, preserving important memories while reducing the overall synaptic load (Tononi and Cirelli 2006). According to the active system consolidation theory, memories are initially stored in the hippocampus and then gradually transferred to the neocortex for long‐term storage during sleep (Diekelmann and Born 2010). This involves a reactivation of recently encoded memories, thereby strengthening and stabilizing them. Frequent sleep in infants may be necessary due to the immaturity of their brain structures, which may require frequent transfer of newly encoded memories to the long‐term storage. From this perspective, changes in sleep behavior may also indicate brain maturation processes (Spencer and Riggins 2022).

Now that seminal findings have shown that sleep supports memory consolidation even in early life, the next step is to investigate the circumstances under which sleep becomes effective. Replications with different tasks and across various age‐groups are essential to test the robustness of these phenomena and to gain a better understanding of the mechanisms underlying the relationship between sleep and memory in infancy. This is especially important in developmental science, where replication helps to confirm the reliability of research findings (Frank et al. 2017). Therefore, the first objective of the present study was to replicate the beneficial effect of a post‐encoding nap on memory in 15‐ and 24‐month‐old infants using a deferred imitation paradigm (research question 1, RQ1). Deferred imitation procedures are a widely used method, which is assumed to serve as a non‐verbal measure of declarative memory in infants (Hayne 2004).

Not all memories encoded during wakefulness undergo sleep‐dependent memory consolidation. For adults, Stickgold and Walker (2013) proposed that sleep‐dependent memory consolidation might be a selective process favoring those memories that are tagged as “relevant.” Memories are initially selected based on the importance ascribed to them at the time of their formation, and these importance markers are then utilized during sleep (Stickgold and Walker 2013). In adults, the personal value attributed to learned material (van Rijn et al. 2017), the anticipation of being tested (van Dongen et al. 2012), or the prospect of monetary rewards (Fischer and Born 2009) can serve as relevance tags, thereby facilitating sleep‐dependent memory consolidation. Preliminary evidence exists for selective sleep‐dependent memory benefits in infants. In Konrad et al. (2019), 15‐ and 24‐month‐old infants participated in a deferred imitation task in which an experimenter demonstrated target actions with unfamiliar objects that were either relevant or irrelevant for achieving the goal of getting access to an attractive object. Half of the infants napped after the demonstration session (nap condition) while the others stayed awake during the subsequent 4 h (no‐nap condition). All infants were tested for their ability to reproduce the target actions after a 24‐h delay. The results revealed a differential impact of sleep on memory consolidation. Whereas infants in the no‐nap condition exhibited faithful reproduction of the demonstrated order (irrelevant action, then relevant), infants in the nap condition failed to reproduce the actions in the demonstrated order. However, contrary to the hypothesis, no significant difference was observed in the reproduction of only relevant actions between the two conditions. Potentially, in that study the differentiation in actions that were relevant versus irrelevant might not have been salient enough to trigger selectivity in sleep‐dependent memory consolidation.

More broadly, it is still unclear which memories might receive relevance tags in infancy as cues of relevance may differ from those that signify future significance to adults. For infants, relevance cues could differ from those relevant for adults due to infants' unique developmental niche (e.g., little prior knowledge and no capacity for future‐directed thinking). We sought to test the effect of cues that might specifically indicate relevance to infants. One “relevance cue” could be the presentation mode of a potential learning experience. Despite infants' ability to learn and remember information delivered on screens (see Barr 2013; Rusnak and Barr 2020; for a review), many studies have also demonstrated that infants' performance when learning and remembering screen‐based information lags behind that of real‐live interactions (e.g., Barr 2010; Barr and Hayne 1999; Brito et al. 2012; Strouse and Samson 2021). Several explanations of this so‐called “transfer deficit” have been discussed, although none of the approaches alone can explain the effect (see Barr and Kirkorian (2023) and Strouse and Samson (2021) for review). Due to the perceptual impoverishment of 2D‐video presentations, fewer cues are available for infants to facilitate memory retrieval after a delay. Moreover, it also leads to a mismatch between perceptual cues encoded from the video demonstration and perceptual characteristics present “in the real world” (Barr 2010, 2013). This complicates memory retrieval particularly for infants because their memory processes are highly specific (Hayne and Herbert 2004) and memory retrieval is vulnerable to even minor changes in cues between encoding and test. Second, understanding of screen content as a representation for actually existing entities (i.e., their symbolic thinking) develops throughout infancy and early childhood (Troseth 2010). Lastly, and most importantly for this study, infants are predisposed to learn from contingent responsive social interactions (Csibra and Gergely 2009; Kuhl 2009), whereby caregivers are an important reference for what is of relevance (Harris and Corriveau 2011). Thus, the transfer deficit is attributed to infants' inability to perceive media content as relevant due to a lack of contingent social reactions typical for educational in‐person interactions (Troseth et al. 2006). In this sense, caregivers can mitigate the transfer deficit by embedding screen content in a social interaction (i.e., caregiver scaffolding, Seehagen and Herbert 2010). For example, Strouse and Ganea (2021) found that 30‐month‐olds benefited from scaffolding when learning labels for objects from a screen (see also Barr and Wyss 2008). A recent meta‐analysis that included 17 studies revealed a small, but significant positive association of adults interactively co‐using digital media with 0 to 6‐year‐olds and learning outcomes (Taylor et al. 2024).

The effect of post‐encoding sleep remains largely unexplored in the context of the transfer deficit. So far, one study has provided preliminary evidence that screen‐based memories for target actions in a deferred imitation task may also benefit from post‐encoding sleep in late infancy (Hermesch et al. 2024). In that study, infants watched two videos each showing an adult model three target actions. One video was accompanied by caregiver scaffolding such that the caregiver commented on the model's actions, whereas the other video was not. Infants were randomly assigned to a nap or a no nap condition. Reproduction of target actions was assessed 24 h later. Twenty‐four‐month‐olds exhibited retention of the target actions, whereas 15‐month‐olds did not. Furthermore, in the 24‐month‐old group, temporal recall of the target actions was significantly enhanced by sleep, but not by caregiver scaffolding.

The present study extends this line of research by investigating whether sleep differentially benefits memory consolidation for actions learned through live demonstrations compared to video demonstrations in 15‐ and 24‐month‐old infants. Manipulation of presentation mode was based on literature indicating that perceived relevance of televised content for real‐life is lower than perceived relevance of socially contingent interactions in infants, despite existing capacities for successful encoding of both (Troseth et al. 2006). Manipulation of scaffolding was based on literature indicating that adult–child interactive co‐use of screen media supports 0 to 6‐year‐olds in their learning (Taylor et al. 2024) and that scaffolding would increase the relevance of the video‐based information. Based on these findings, we sought to investigate whether making this information more relevant to them would lead to sleep‐dependent memory consolidation. There were two main reasons for selecting these age‐groups: (a) Ecological relevance: Parents more intentionally provide media experiences in the second year of life than in the first year of life (Rideout 2017), and (b) numerous transfer deficit studies have been conducted during the second year of life (for review, see Barr and Kirkorian 2023). Consequently, identifying strategies to mitigate this phenomenon is of particular significance in this age group. By comparing data from the current live demonstration experiment to the previously completed video experiment (research question 2, RQ2), we aimed to gain a deeper understanding of how the presentation mode of learning experiences and subsequent sleep influence memory consolidation in infancy. If televised content is perceived as irrelevant, it should be less likely to undergo sleep‐dependent memory consolidation and a potential memory benefit of post‐encoding sleep should be less pronounced. To allow cross‐experiment comparisons, and thereby to answer RQ2, we used the exact same procedures in the live experiment as in the video experiment conducted by Hermesch et al. (2024), including the within‐subject manipulation of caregiver scaffolding.

We tested two following hypotheses: First, in RQ 1 (Replication of the beneficial effect of a post‐encoding nap) the primary hypothesis was that in both age groups, infants in the nap condition would reproduce a higher number of target actions than infants in the no‐nap condition. Moreover, we expected that infants in the nap and the no‐nap condition would show retention of the target actions. That is, infants in these conditions will perform a significantly higher number of target actions compared to infants in the age‐matched control conditions. We also expected a main effect of age with infants aged 24 months reproducing a greater number of target actions than infants aged 15 months. The impact of caregiver scaffolding was investigated through an exploratory approach.

In RQ 2 (cross‐experiment comparison), we predicted that a memory benefit of post‐encoding sleep in the nap condition (vs. the no‐nap condition) would be more pronounced when infants saw a live (vs. video) demonstration (i.e., a presentation mode × sleep condition interaction). We also expected to find a transfer deficit: infants who watched a live demonstration were expected perform a higher number of target actions than infants who watched a televised demonstration of target actions. Moreover, we predicted that 24‐month‐old infants would remember more target actions than 15‐month‐old infants across both experiments.

## 2 Methods

We declare that we did not use any artificial intelligence‐generated content (AIGC) tools, such as ChatGPT or other large language model (LLM)‐based tools, in the development of any part of this manuscript.

### 2.1 Design and Participants

Regarding RQ 1, the present study examined the effect of sleep on infants' imitation performance from a live demonstration. The study can be described as a mixed experimental design with age (15, 24 months) and condition (nap, no‐nap, baseline) as between‐subject factors and presence of caregiver scaffolding as a within‐subject factor. For the cross‐experiment comparison (RQ 2), the data were compared to those of a previously published video experiment (see Hermesch et al. 2024, for a more detailed description). Presentation mode (live, video) and sleep condition (nap, no‐nap) were between‐subject factors, whereas caregiver scaffolding was a within‐subject factor.

**FIGURE 1:**
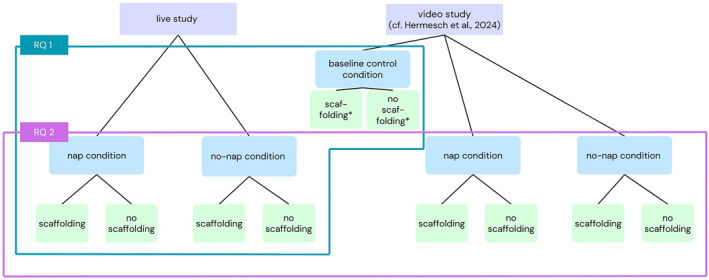
Overview of the design within each study and the allocation of subsamples per research question. In both studies, data for each cell of design was collected in 15‐ and 24‐month‐old infants. *The levels of scaffolding (yes vs. no) in the baseline control condition are not meaningfully interpretable. Average scores across both imitation tasks were used for analyses.

In both studies, infants in the nap condition napped for ≥ 30 uninterrupted minutes within 4 h after the demonstration session. Infants in the no‐nap condition were required to stay awake for ≥ 4 h after the demonstration session (i.e., 0 actigraphy‐detected minutes of sleep). Figure 1 illustrates the allocation of subsamples for RQ1 and RQ2. Procedures and analyses were preregistered (https://osf.io/fkv2t).

#### 2.1.1 Sample 1: Live Demonstration

A power analysis using G*Power (Faul et al. 2009) was conducted in preparation for the video study (for details see: https://osf.io/nehf5, section sample size rationale). Aiming for similar sample sizes per group across conditions, we adopted a sample size of *n* = 17 per sleep condition (nap, no‐nap) and age group from the video study. The sample of the live experiment comprised *n* = 68 infants, distributed equally between two age‐groups of 15 and 24 months (± 1 month). The mean age of the 15‐month‐olds was 469 days (SD = 12.2; 41% female). The mean age of the 24‐month‐olds was 735 days (SD = 12.3; 43% female). Infants were randomly assigned to a nap or a no‐nap condition. Infants in the nap condition were required to sleep ≥ 30 min within 4 h after demonstration of the target actions. Infants in the no‐nap condition were required to stay awake ≥ 4 h after the demonstration of target actions. Participants were recruited via the infant and child subject database of the Faculty of Psychology at Ruhr University Bochum, Germany. A research assistant contacted the caregivers of healthy full‐term infants in the respective age ranges who had previously signed up for the database and informed them about the study procedures. If caregivers were interested, study appointments were scheduled to match the requirements of the nap or no‐nap condition based on caregiver‐reported infant sleep schedules. Consequently, the first appointment in the nap condition was scheduled shortly before infants were expected to naturally take a nap as reported by their caregivers. The first appointment in the no‐nap condition was scheduled shortly after infants typically woke up in the morning or after a nap. All participating families received a gift (e.g., a small toy or a picture book) and a certificate. The majority (65%) of the caregivers stated that German was the only language spoken in the household. Fifteen percent of the infants were exposed to at least one more language in addition to German at home. For one fifth of the infants (*n* = 14) caregivers chose not to answer additional demographic questions. Infants from families with a higher educational degree were overrepresented in this sample, with 53% of caregivers holding a university degree or higher. All participating families lived in the metropolitan area of Bochum in Germany. Twenty‐four additional infants were tested, but were not eligible for main analyses because their sleep behavior was inconsistent with assigned condition, that is either sleep within 4 h after the first appointment in the no‐nap condition or no sleep in the nap condition (*n* = 7), there was no valid actiwatch data (*n* = 6), they did not touch the stimuli (*n* = 4), the caregiver interfered (*n* = 3), due to technical error (*n* = 2) or illness‐related cancellation of the test session (*n* = 2). Data were collected from November 2022 to December 2023.

#### 2.1.2 Sample 2: Video Demonstration

An a priori power analysis was conducted using G*Power (Faul et al. 2009) assuming a medium effect size (*f* = 0.25) while the power (1 − *β*) was set to 0.90 and the α‐level was set to 0.05 to test the key prediction of a two‐way interaction between condition (nap vs. no nap) and scaffolding (scaffolding, no scaffolding). The result for the total sample was *N* = 102. As reported in Hermesch et al. (2024), the final sample for the video experiment consisted of *n* = 34 infants aged 15 months and *n* = 35 infants aged 24 months, also distributed among both age groups and within each age group, equally distributed among a nap and a no‐nap condition (see Hermesch et al. (2024), for a more detailed description). As in the live demonstration sample, most caregivers stated that German was the only spoken language in their household (58%). Nineteen percent of caregivers reported that at least one other language apart from German was spoken in their household. Again, about one fifth of the caregivers (*n* = 23) chose not to provide additional demographic data in an online questionnaire. In this sample families with higher educational degrees were again overrepresented, with 56% of the caregivers holding a university degree or higher. Participating families also lived in the metropolitan area of Bochum, Germany. Within the video study *n* = 36 additional participants were tested in age‐matched baseline control conditions (*n* = 17 infants aged 15 months and *n* = 19 infants aged 24 months). Recruitment and random allocation to sleep conditions was carried out as described above. Data were collected from September 2021 to October 2022.

### 2.2 Materials

#### 2.2.1 Deferred Imitation Tasks

To assess declarative memory consolidation, each infant completed two well‐established deferred imitation tasks (e.g., Barr et al. 2007; Barr and Hayne 1999; J. Herbert and Hayne 2000a, 2000b). These tasks were equivalent in the live and the video study. In both tasks, infants were required to assemble a toy in three steps, one of which was a rattle toy and the other one a wooden rabbit toy. The stimuli and the target actions to assemble each toy are depicted in Table 1.

**TABLE 1:** Stimulus sets and target actions of imitation tasks.

Stimulus set	Target action 1	Target action 2	Target action 3
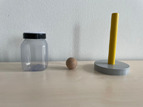	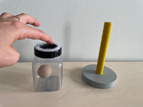	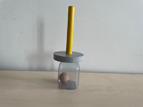	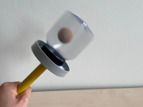
Rattle task	Push wooden bead through diaphragm into jar	Place stick on jar attaching with Velcro	Shake stick to make noise
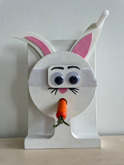	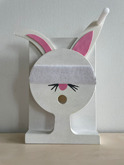	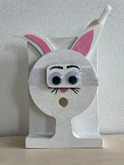	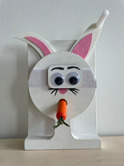
Animal task	Pull handle in circular motion to raise ears	Place eyes on face attaching with jar	Put carrot in rabbit's mouth

#### 2.2.2 Actigraphy

In both studies, actiwatches (Micro Motionlogger, Ambulatory Monitoring Inc.) were used to monitor sleep‐wake behavior between the demonstration and the test session 24 h later. The wristwatch‐like devices record frequency, intensity, and duration of movements. Thus, actigraphy is a non‐invasive and convenient method to objectively measure sleep behavior in naturalistic settings (Ancoli‐Israel et al. 2003). Caregivers were also asked to keep a sleep diary for the monitored period to control for potential artifacts in movement data.

### 2.3 Procedure

#### 2.3.1 Sample 1. Live Demonstration

Study participation in the live demonstration experiment involved two home visits scheduled 24 h apart. During the first home visit, informed consent was obtained from the caregiver. After a short familiarization phase, the experimenter asked the caregiver to sit at the family's table with the infant on the lap or to seat the infant in a highchair while the experimenter took a seat opposite the infant. Then the experimenter demonstrated the target actions three times in succession for both tasks one after another. For one set of stimuli, the experimenter asked the caregiver to scaffold encoding by reading out a standardized script (e.g., “Look, she has made something!”; cf. Barr and Wyss 2008). Verbal labels for the stimuli or actions were not provided in the scaffolding condition (i.e., infants received empty narration). In general, caregivers were instructed not to comment any further on what the infant observed during the demonstration. The order of demonstrations and the assignments of imitation tasks to the scaffolding and no scaffolding conditions was counterbalanced across conditions. After the demonstration session, the actiwatch was attached to the infant's ankle.

Infants' reproduction of target actions was tested 24 h later during the second home visit. Again, the caregiver and the experimenter took a seat at the table. Then the experimenter placed the first set of stimuli within the infant's reach. She said, “Look. Now it is your turn.” The infant had 90s to reproduce the target actions from first touching the stimuli. The process was subsequently carried out with the second set of stimuli. The presentation order for the stimuli was identical in the demonstration and the test session. Throughout the test phases, no verbal labels were used for the stimuli or the actions. Both the demonstration and the test session were video recorded.

#### 2.3.2 Sample 2. Video Demonstration

Due to the Covid‐19 pandemic, the demonstration and test phases were conducted via video chat appointments also scheduled 24 h apart. Instead of a live demonstration, target actions were demonstrated by a female model on a pre‐recorded video on an experimenter provided tablet. Due to the videochat setting, experimenters instructed the caregivers to start the videos for demonstration and to place the stimuli within the infant's reach during the test phase, respectively (for a detailed description, see Hermesch et al. 2024).

#### 2.3.3 Baseline Control Condition

Data collection for the baseline control condition was also conducted via video chat appointments as part of the video study. Without prior demonstration, caregivers were first instructed to place both sets of stimuli within infants' reach one after another and then infants had 90s to interact with each stimulus. Subsequently, infants from the baseline control condition watched both video demonstrations of the target actions and were then tested for their immediate imitation of target actions on both stimuli, which is not relevant to any of the research questions addressed in this article and will not be discussed further (for details, see Hermesch et al. 2024).

### 2.4 Data Coding

Movement data collected by the actiwatches were analyzed in Action‐W software (Ambulatory Monitoring Inc.; Ambulatory Monitoring 2023) in 1‐min epochs using zero‐crossing mode. Sleep‐wake behavior was scored with the aid of an algorithm developed by Sadeh et al. (1994) for adults and infants aged > 1 year. Only when sleep diaries suggested that sleep scoring might be biased due to potential artifacts (e.g., external movement during a car ride) were caregiver‐reported sleep and wake up times given preference.

Infants' visual attention to the experimenter demonstrating the target actions was coded frame by frame using Mangold Interact (Version 18.03.20; Mangold International, 2018) to assess if looking time was above the preregistered criteria of 50%.

The same coding scheme for the deferred imitation tasks was used in both the video demonstration and the live demonstration experiments. The imitation score was coded for each video‐recorded test session and quantified the number of target actions reproduced per set of stimuli. One point was given for each target action, regardless of order (range: 0–3 points). Additionally, a sequence score captured if infants imitated the target actions in the demonstrated order (range: 0–2 points) and we coded the latency to first target action (time interval between first touching the stimuli and the first imitated target action). Thirty percent of the test sessions were coded by a second independent coder. Interrater reliability can be interpreted as high (*к* = 0.94 on average). We also counted the number of prescribed phrases read out aloud to check caregiver compliance in the scaffolding condition. To be eligible for our analyses, caregivers had to read out the required minimum of three out of six phrases. All caregivers in the final sample met this criterion.

### 2.5 Statistical Analyses

To analyze infants' imitation performance from a live demonstration as a function of age, condition and presence of scaffolding (RQ 1) and imitation performance as a function of presentation mode, age, condition and scaffolding (RQ 2) we conducted two mixed analyses of variances (ANOVAs) respectively. In the live study, we expected infants in the nap condition would reproduce significantly more target actions than infants in the no‐nap condition (main effect of group). Moreover, we expected infants in both sleep conditions and at both ages to outperform their age‐matched baseline control condition. We also predicted a main effect of age with 24‐month‐olds performing more target actions than 15‐month‐olds. To reduce complexity in the design and as comparisons were already made within each study (live, video), we decided to remove the baseline control condition from the cross‐experiment analyses. Our main predictions were that we would find a main effect of presentation mode and a presentation mode × sleep condition interaction. Specifically, we expected the beneficial effect of a post‐encoding nap on memory performance to be more pronounced when infants encoded from a live (vs. video) demonstration. Again, we expected to find a main effect of age such that 24‐month‐olds would reproduce a higher number of target actions than 15‐month‐olds.

## 3 Results

### 3.1 Descriptive Results

Visual attention to the demonstration was high (mean looking time: 95% (SD = 9%)). Table 2 provides information on the timing of the study appointments and infants' sleep from the live study. Comparisons with the video study revealed that timing of study appointment in the no‐nap condition significantly differed between studies in both age groups (see Appendix A, Tables A1 and A2), but, as in the video study, imitation performance was unrelated to test timing (*ρ* = 0.05, *p* = 0.658). Sleep duration within 4 h from encoding and latency to the next sleep episode within sleep conditions did not differ between studies (see Appendix A, Table A1). Caregiver‐reported 24‐h sleep duration in the two weeks preceding the study appointments did not differ between studies (*t*(103) = 0.00, *p* = 0.997). Descriptive results on infants' imitation performance as a function of presentation mode, age, condition and scaffolding are displayed in Table 3.

**TABLE 2:** Mean sleep and test timing as a function of age and sleep condition in the live study.

Age group	Sleep condition	Mean time of test in hh:mm (SD)	Mean sleep duration within 4 h after encoding in min (SD)	Mean latency to next sleep episode in min (SD)
15 months	Nap	10:31 (00:44)	73.29 (32.94)	79.41 (51.44)
	No‐nap	13:23 (02:37)	0 (0)	309.41 (56.15)
24 months	Nap	10:26 (01:00)	88.41 (33.85)	89.59 (50.19)
	No‐nap	15:06 (00:24)	0 (0)	300.76 (32.05)

**TABLE 3:** Mean number and standard deviation of produced target actions in the video and the live study.

		Presentation mode			
		Live		Video	
Condition	Scaffolding	15 months	24 months	15 months	24 months
Nap	Scaffolding	*M* = 1.29	*M* = 2.88	*M* = 0.65	*M* = 2.18
		SD = 0.92	SD = 0.33	SD = 0.79	SD = 1.13
	No scaffolding	*M* = 1.88	*M* = 2.71	*M* = 0.88	*M* = 2.35
		SD = 0.86	SD = 0.59	SD = 0.93	SD = 0.93
No‐nap	Scaffolding	*M* = 1.06	*M* = 2.88	*M* = 0.53	*M* = 1.76
		SD = 0.97	SD = 0.33	SD = 0.94	SD = 1.15
	No scaffolding	*M* = 1.41	*M* = 2.53	*M* = 0.88	*M* = 1.76
		SD = 1.12	SD = 0.80	SD = 0.99	SD = 0.97
Baseline	(Averaged across levels of scaffolding)			*M* = 0.41	*M* = 0.92
				SD = 0.32	SD = 0.24

### 3.2 Research Question 1: Effect of a Post‐Encoding Nap on Memory

#### 3.2.1 Number of Target Actions Recalled

The first hypothesis that infants who napped soon after encoding would reproduce a significantly higher number of target actions as compared to infants who stayed awake was not confirmed. We tested this by conducting a 2(age: 15 months, 24 months) × 3(condition: nap, no‐nap, baseline control) × 2(scaffolding: yes, no) mixed ANOVA on the number of reproduced target actions in the live study with repeated measures on scaffolding. There was a main effect of age (*F*(1,98) = 91.10, *p* < 0.001, $\eta^{2}g$ = 0.32), a main effect of sleep condition (*F*(1,98) = 74.43, *p* < 0.001, $\eta^{2}g$ = 0.43), an age × condition interaction (*F*(2,98) = 6.74, *p* = 0.002, $\eta^{2}g$ = 0.07) and an age × scaffolding interaction (*F*(2,98) = 10.13, *p* = 0.002, $\eta^{2}g$ = 0.03). Significant interaction effects were followed up with Tukey‐corrected pairwise contrasts. Pairwise contrasts disentangling the age × condition interaction are depicted in Table 4. The pairwise comparison of the nap and the no‐nap condition was not significant at both ages.

**TABLE 4:** Pairwise comparison to follow up significant age × condition interaction.

Dependent variable	Comparison	15 months	24 months
Number of reproduced target actions	Baseline—Nap	*M*_Diff_ = −1.18, *p* < 0.001	*M*_Diff_ = −1.87, *p* < 0.001
	Baseline—No‐nap	*M*_Diff_ = −0.82, *p* < 0.001	*M*_Diff_ = −1.78, *p* < 0.001
	Nap—No‐nap	*M*_Diff_ = 0.35, *p* = 0.170	*M*_Diff_ = 0.09, *p* = 0.893
Sequence of reproduced target action	Baseline—Nap	*M*_Diff_ = −0.47, *p* = 0.007	*M*_Diff_ = −1.20, *p* < 0.001
	Baseline—No‐nap	*M*_Diff_ = −0.41, *p* = 0.020	*M*_Diff_ = −0.91, *p* < 0.001
	Nap—No‐nap	*M*_Diff_ = 0.06, *p* = 0.919	*M*_Diff_ = 0.29, *p* = 0.129

The second hypothesis, that infants in both sleep conditions (nap, no‐nap) would exhibit retention of target actions after 24 h when they were demonstrated live was confirmed. Pairwise comparisons revealed that infants in both sleep conditions outperformed the age‐matched infants in the baseline control condition (see Table 4).

**FIGURE 2:**
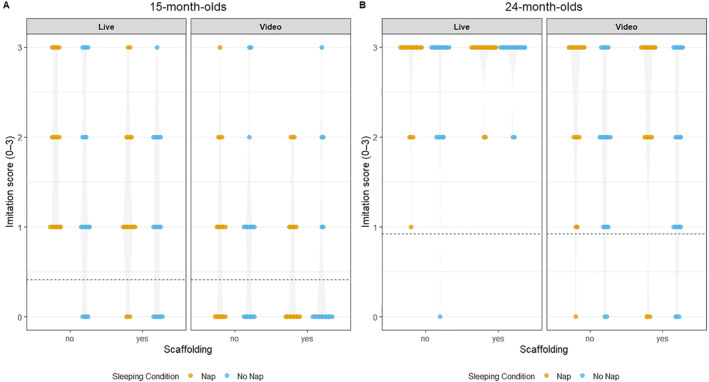
Beeswarm plot for cross‐experiment comparison of imitation scores.

The third hypothesis, that 24‐month‐olds would produce a higher number of target actions than 15‐month‐olds was confirmed by a main effect of age in the above mentioned ANOVA. A graphic impression of the age × condition interaction (see Figure 2) shows that the difference between baseline and sleep conditions was larger in 24‐month‐olds than in 15‐month‐olds. This can be interpreted as an age‐related increase in memory performance. Surprisingly, there was a disruptive effect of caregiver scaffolding in 15‐month‐olds: Pairwise contrasts to follow up the age × scaffolding interaction revealed that 15‐month‐olds reproduced significantly fewer target actions in the imitation task with caregiver scaffolding as compared to without caregiver scaffolding (*M*_Diff_ = −0.314, *p* = 0.005). In 24‐month‐olds imitation performance did not differ as a function of caregiver scaffolding (*M*_Diff_ = 0.176, *p* = 0.105).

#### 3.2.2 Temporal Recall

A mixed ANOVA on the sequence of reproduced target actions mainly supported previously reported results on the number of reproduced target actions: Again, there were significant main effects of age (*F*(1,98) = 56.56, *p* < 0.001, $\eta^{2}g$ = 0.23) and condition (*F*(2,98) = 35.63, *p* < 0.001, $\eta^{2}g$ = 0.27) and a significant age × condition interaction (*F*(2,98) = 6.40, *p* = 0.002, $\eta^{2}g$ = 0.06). Post hoc pairwise comparisons confirmed that in both the nap and the no‐nap condition infants did not only outperform the age‐matched baseline control group in the number of reproduced target actions, but also imitated the target actions in the demonstrated order above chance level (see Table 4). Twenty‐four‐month‐olds had a significantly higher sequence score and the difference between baseline and sleep conditions was descriptively more pronounced in the older age group suggesting that memory performance increased with age.

A mixed ANOVA on the latency to first target action revealed only a main effect of age (*F*(1, 52) = 15.62, *p* < 0.001, $\eta^{2}g$ = 0.16). Twenty‐four‐month‐olds produced the first target action significantly faster than 15‐month‐olds. The sample size for this analysis was reduced since meaningful values for this variable could only be assigned to infants who performed at least one target action in both imitation tasks.

For correlative analyses infants were also included if their sleep behavior was not in accordance with the assigned sleep condition. Results are displayed in Table 5. Overall, infants' sleep behavior within 4 h after demonstration and in the subsequent night was unrelated to imitation performance. Only a longer night's sleep was associated with a higher number of reproduced target actions in 24‐month‐olds (*ρ* = 0.46, *p* = 0.008).

**TABLE 5:** Correlations between sleep behavior and imitation performance.

		15 months	24 months
Latency to next sleep episode (in min)	*M* (SD)	186.95 (124.69)	195.18 (114.92)
	*ρ*_imi_	−0.18	−0.35
	*ρ*_*s*eq_	0.03	−0.37
Sleep duration within 4 h after encoding (in min)	*M* (SD)	36.45 (41.40)	42.94 (50.49)
	*ρ*_imi_	0.18	0.15
	*ρ*_*s*eq_	0.06	0.21
Night‐time sleep duration (in min)	*M* (SD)	479.70 (65.51)	504.60 (64.61)
	*ρ*_imi_	0.02	0.46*, *p* = 0.008
	*ρ*_seq_	−0.07	0.32, *p* = 0.074
Wake minutes after sleep onset	*M* (SD)	167.10 (66.12)	116.20 (48.84)
	*ρ*_imi_	0.12	0.01
	*ρ*_seq_	0.03	−0.05
Sleep efficiency (in %)	*M* (SD)	74.50 (8.58)	81.40 (7.07)
	*ρ*_imi_	−0.11	0.16
	*ρ*_seq_	−0.02	0.12
Longest wake episode (in min)	*M* (SD)	65.94 (42.71)	57.94 (33.18)
	*ρ*_mi_	0.04	−0.22
	*ρ*_seq_	0.13	−0.25

*p < 0.05.

### 3.3 Research Question 2: Cross‐Experiment Comparison

#### 3.3.1 Number of Target Actions Recalled

Although non‐significant in each individual study, when data was combined across studies infants who napped soon after encoding reproduced significantly more target actions as compared to infants who remained awake after encoding. We tested this by drawing on data from both experiments and conducting a 2(presentation mode: live, video) × 2(age: 15, 24 months) × 2(condition: nap, no‐nap) × 2(caregiver scaffolding: yes, no) mixed ANOVA on the number of target actions produced at test. There was a significant main effect of nap condition (*F*(1,129) = 4.60, *p =* 0.034, $\eta^{2}g$ = 0.02).

Contrary to expectations, the beneficial effect of a post‐encoding nap was not more pronounced in infants who encoded the target actions from a live demonstration versus from a televised demonstration, as indicated by the non‐significant presentation mode × sleep condition interaction (*F*(1,129) = 0.005, *p* = 0.821, $\eta^{2}g$ < 0.01).

As expected, we found a significant effect of presentation mode (*F*(1,129) = 37.28, *p* < 0.001, $\eta^{2}g$ = 0.14) indicating that infants reproduced more target actions when they were demonstrated live as compared to a video demonstration.

Again, there was a significant main effect of age (*F*(1,129) = 129.99, *p* < 0.001, $\eta^{2}g$ = 0.36) with a higher imitation score in 24‐month‐olds. In addition, there was a significant age × scaffolding interaction (*F*(1,129) = 4.88, *p* < 0.029, $\eta^{2}g$ = 0.02). Post hoc pairwise comparisons indicated that 15‐month‐olds reproduced significantly fewer target actions in the presence of caregiver scaffolding (*M*_Diff_ = −0.38, *p* = 0.008). There was neither a disruptive nor a protective effect of caregiver scaffolding on memory performance in 24‐month‐olds *w* (*M*_Diff_ = 0.06, *p* = 0.669).

#### 3.3.2 Temporal Recall

The pattern of results in the mixed ANOVA on the sequence of reproduced target actions was similar to the target actions findings. A higher number of target actions were performed in the correct order following the live demonstration than following the video demonstration (main effect of presentation mode *F*(1,129) = 22.99, *p* < 0.001, $\eta^{2}g$ = 0.09). Moreover, there was a main effect of age (*F*(1,129) = 4.24, *p* < 0.001, $\eta^{2}g$ = 0.25), a main effect of sleep condition (*F*(1,129) = 4.24, *p* = 0.041, $\eta^{2}g$ = 0.02) and an age × sleep condition interaction (*F*(1,129) = 4.98, *p* = 0.021, $\eta^{2}g$ = 0.02). Post hoc pairwise comparisons indicated that only in 24‐month‐olds, napping facilitated reproduction of target actions in the demonstrated order (*M*_Diff_ = 0.37, *p* = 0.003). This is likely due to an inability to detect a difference at 15‐months due to overall low sequence scores in this age‐group.

## 4 Discussion

The present study aimed to replicate the findings of previous research on the beneficial effects of a post‐encoding nap on memory consolidation in infants and young children (see Mason and Spencer 2022; Seehagen et al. 2019 for review). The combined analysis of both experiments revealed a significant beneficial effect of a post‐encoding nap on memory consolidation. This finding, while not significant in each individual sample, highlights the importance of considering cumulative evidence to draw conclusions from multiple studies, perhaps particularly in research areas that traditionally feature small sample sizes. Moreover, we explored the role of the presentation mode and caregiver scaffolding in this context. Infants' memory performance after a live demonstration exceeded that of a video demonstration, indicating a transfer deficit in both age groups. An empty narration provided by the caregiver to scaffold learning impeded the reproduction of target actions in 15‐month‐olds, both when the live study was considered individually, and when comparing the two experiments.

In the cross‐experimental comparison, we found that a post‐encoding nap of at least 30 min facilitated declarative memory retrieval after 24 h. Together with other studies investigating the same effect across different tasks and age groups (e.g., Berger and Scher 2017; DeMasi et al. 2021; Friedrich et al. 2015, 2020; Hupbach et al. 2009; Horváth et al. 2015; Kurdziel et al. 2013; Simon et al. 2017; Seehagen et al. 2015), this highlights sleep's supportive role for early memory processes. This finding is consistent with the active systems consolidation theory, which suggests that sleep‐dependent memory transfer from the hippocampus to the neocortex is crucial for long‐term storage. (Diekelmann and Born 2010). However, the effect size in our study was small and the effect was only significant when the data was combined across experiments. This small effect can be attributed to methodological issues within individual studies. In the live study, we observed a ceiling effect in 24‐month‐olds who reproduced 2.75 out of 3 reproduced target actions on average from a live demonstration. On the contrary, there was a floor effect in 15‐month‐olds when imitating from a video demonstration and their memory performance did not exceed baseline levels. It comes as no surprise that the difference between infants who napped and those who stayed awake is least pronounced in these circumstances. In other words, the floor and ceiling effects may have obscured a potential beneficial effect of a post‐encoding nap (see also Petzka et al. 2021) resulting in non‐significant results in each individual study and curtailing the overall effect size. Overall, the pattern of results emphasizes the value of cumulative evidence and larger sample sizes and highlights the challenge of creating memory tasks of appropriate difficulty for different age groups in infancy to prevent ceiling and floor effects.

Given the absence of a presentation mode × sleep condition interaction, we also conclude that memories of information presented on screen and live benefit from post‐encoding sleep to a similar extent. However, this result contradicts our hypothesis that screen‐based memories may not be tagged as relevant and thus benefit to a lesser extent from sleep‐dependent memory consolidation. This hypothesis was prompted by the idea that sleep selectively strengthens memories tagged as relevant (Stickgold and Walker 2013) and that infants may fail to perceive screen content as a relevant information to remember (Troseth et al. 2006). However, our results do not provide evidence that the presentation mode determines the consolidation of recently encoded target actions during sleep by acting as a relevance tag. In addition, embedding screen content in a social interaction as operationalized by caregiver scaffolding did not interact with infants' sleep status and thus did not emerge as a relevance cue for sleep‐dependent memory consolidation either. As discussed in Hermesch et al. (2024) the difference between both scaffolding conditions might have been too small in terms of the social context of viewing to elicit selectivity in sleep dependent memory consolidation of the presented content. Potentially, the presence of the caregiver and the co‐viewing of the video demonstrations constituted a context that signaled “relevance,” regardless of caregiver vocalizations. This explanation can be extended to the entire comparison of the video and the live study. In general, watching the demonstration video was embedded in an online study appointment, thus a minimum of social interaction could not be avoided. In everyday life, a relevant proportion of early media exposure happens outside of any direct caregiver involvement (Ewin et al. 2021; Levine et al. 2019). The question remains open if infants' solitary screen viewing in a no‐scaffolding condition would lead to the same results. Future research could include a solo viewing condition to test this hypothesis. Provided that sleep‐dependent memory consolidation is a selective process in infants (for a critical discussion about the robustness of the effect in adults see Cordi and Rasch 2021), possible relevance tags that apply to infants were not disentangled in the current study (Hermesch et al. 2024; Konrad et al. 2019).

Our cross‐experiment comparison revealed a significant effect of presentation mode, with live demonstrations leading to better memory performance than screen‐based demonstrations in the second year of life. Whereas 15‐month‐olds did not exhibit retention of target actions after 24 h from a video demonstration, they did from a live presentation, irrespective of sleep status. Imitation performance of 24‐month‐olds approached ceiling levels. From additional analyses with infants in the baseline control condition reported in Hermesch et al. (2024) it was evident that 15‐month‐olds showed immediate imitation of target actions above baseline levels from the same video demonstration, indicating that they were able to encode the target actions. However, screen‐based memories were less stable and could not be retrieved after a 24‐h delay. Several studies have documented poorer memory retrieval of televised target actions in deferred imitation tasks (e.g., Barr and Hayne 1999; Barr et al. 2007; Barr and Wyss 2008). It is also possible that, in addition to a retrieval and transfer challenge, encoding from a video demonstration results in a poorer memory trace, which degrades more quickly over time (Barr and Kirkorian 2023). With increasing age, memory processes in infants increase in flexibility (Hayne 2004) and infants are more likely to master the transfer from cues encoded from a 2D‐screen to later in vivo situations (Barr and Kirkorian 2023; Strouse and Samson 2021). This is in line with our result that 15‐month‐olds failed in the critical comparison with the baseline control condition to infer memory retrieval in the video study, while 24‐month‐olds passed it. However, even at the age of 24 months, memory performance from a video demonstration was significantly poorer as compared to a live demonstration. In sum, retrieving target actions from a video demonstration after a delay was cognitively more demanding due to fewer and more divergent cues as compared to a live demonstration, resulting in poorer memory performance of screen‐based target actions in both age groups.

Another factor that unexpectedly added to the cognitive demands within the memory processes at least in younger infants was the presence of caregiver scaffolding. In the video study, we expected caregiver scaffolding to facilitate sleep‐dependent memory consolidation and later memory retrieval by highlighting the relevance of the content. Contrary to our expectations, there was no significant effect of caregiver scaffolding, instead there was a descriptive trend toward a poorer performance of 15‐month‐olds in the scaffolding condition. Even more unexpected was the pattern of results in the live study. We found that caregiver scaffolding significantly hindered memory performance in 15‐month‐olds when only looking at the live data, and across both experiments. In our manipulation, scaffolding was restricted to an empty narration, thus it did not contain any valuable information that helps to understand how to assemble the toys. We chose empty narration to investigate the function of caregiver scaffolding without other confounding factors such as additional verbal cues. While the facilitative effect of scaffolding in general has been well documented across studies (Clegg and Legare 2017; Hayne and Herbert 2004; J. S. Herbert 2011; Simcock et al. 2011), there seems to be a consensus that an empty narration does not facilitate learning to the same extent as a narration that provides additional valuable information, for example by labeling and describing the objects and actions presented (Barr and Wyss 2008; Hayne et al. 1997; Hayne and Herbert 2004; Seehagen and Herbert 2010; Taylor et al. 2016). Research by Taylor et al. (2016) with 12‐month‐olds demonstrated that providing meaningful verbal labels is key to scaffolding effectiveness. They found that verbal labels from an unknown language (Chinese labels for English‐speaking infants) or empty narration did not aid memory retrieval, suggesting that scaffolding's benefit comes from meaningful input, not just from guiding attention. Similarly, Zack et al. (2013) described that when meaningful and nonsense verbal input during demonstration was too complex for 15‐month‐olds, the performance in a 2D‐3D transfer task was constrained. The authors argued that at this age, the competing cognitive demands of simultaneously processing verbal input while managing the transfer of information across dimensions exceeded attentional capacities (see also Barr and Kirkorian 2023 for additional information). This supports our conclusion that at this developmental stage, caregiver scaffolding that lacks relevance or is too complex can increase cognitive load and may interfere with, rather than support, memory encoding.

As discussed, a major limitation of the present study is that we were not able to include a video condition that resembles infants' solitary digital media use without any caregiver involvement. In Hermesch et al. (2024) we outlined that laboratory settings could offer the opportunity to implement more stringent manipulations in terms of a no interaction video control condition. Moreover, our interpretations on the effects of scaffolding are limited to a standardized empty narration provided by the caregiver. In a recent meta‐analysis, Taylor et al. (2024) identified the need for better operationalization of co‐use and scaffolding during early digital media use to disentangle how and what supports infants' and young children's learning. When investigating sleep‐mediated memory effects, studies with infants should be carefully designed with respect to task difficulty perhaps testing with more items to avoid ceiling and floor effects. In general, more research is needed that systematically addresses the role of task difficulty for sleep‐dependent memory consolidation in infants. This could be achieved for example by varying the number of demonstrations as a function of age group and targeted task difficulty (e.g., Barr et al. 1996) or to assure that individual encoding levels have reached a pre‐defined threshold (see Petzka et al. 2021 for a study with adults). Moreover, our results are limited to behavioral evidence of sleep‐mediated memory effects. Future studies could investigate whether slow‐wave sleep during post‐encoding sleep or sleep spindle activity is related to subsequent memory performance, as some studies have shown in infants (e.g., Friedrich et al. 2015, 2020; Horváth et al. 2018; Kurz et al. 2024). A more rigorous test would involve a targeted memory reactivation study in which a sound is paired with the learning event during wakefulness and then replayed during specific sleep stages (see Carbone and Diekelmann 2024, for a review; Rasch et al. 2007). If infants who heard the sound during both learning and slow‐wave sleep exhibit better learning than those who either did not hear the sound or heard it during other sleep stages, this would strongly support the active systems consolidation theory. Lastly, the comparably short estimated night‐time sleep duration based on our actigraphy data questions the validity of actigraphic sleep assessment in samples with a high prevalence of co‐sleeping. This should not limit the interpretability of our results on post‐encoding day‐time naps, where co‐sleeping is typically less common and actigraphy data and caregiver reports show a high level of agreement. However, it poses a challenge to future studies that aim to disentangle the unique contributions of a nap soon following encoding and the subsequent night‐time sleep (Seehagen et al. 2019) using established ambulatory sleep assessment methods.

## 5 Conclusion

It has only been during the last decade that researchers has begun to better understand the role of sleep in early cognitive processes using experimental approaches. Over the same period, the prevalence of digital devices in the lives of infants and young children has also dramatically increased. The present study sheds light on how sleep strengthens memories encoded from social interactions but also from screen devices. From a theoretical and applied perspective, it is essential to better understand how learning from digital media can be boosted in early childhood and how memories of digital content are processed during sleep.

## Author Contributions

**Neele Hermesch:** conceptualization, writing – original draft, investigation, formal analysis, methodology. **Sabine Seehagen:** supervision, resources, project administration, conceptualization, funding acquisition, methodology, writing – review and editing. **Rachel Barr:** conceptualization, writing – review and editing, methodology, supervision. **Jane S. Herbert:** conceptualization, writing – review and editing, supervision, methodology, supervision. **Carolin Konrad:** supervision, conceptualization, writing – original draft, writing – review and editing, funding acquisition, resources, methodology, project administration.

## Ethics Statement

This study was approved by the local Ethics Committee of the Faculty of Psychology, Ruhr University Bochum.

## Conflicts of Interest

The authors declare no conflicts of interest.

## Data Availability

The data that support the findings of this study are openly available in OSF Storage at https://osf.io/vtwf2.

## Appendix A

**TABLE A1:** ANOVA results for timing of study appointments, sleep duration and latency to next sleep episode as a function of experimental condition and study.

	Timing of study appointments			Sleep minutes within 4 h			Latency to next sleep episode		
Effect	*F* ratio	df	$\eta_{G}^{2}$	*F* ratio	df	$\eta_{G}^{2}$	*F* ratio	df	$\eta_{G}^{2}$
Study	0.34	1, 128	0.00	0.95	1, 128	0.01	0.16	1, 128	0.00
Age group	0.71	1, 128	0.01	4.41*	1, 128	0.03	0.01	1, 128	0.00
Condition	189.85**	1, 128	0.60	423.22**	1, 128	0.77	499.92**	1, 128	0.80
Study × age group	6.72*	1, 128	0.05	0.05	1, 128	0.00	0.00	1, 128	0.00
Study × condition	0.92	1, 128	0.01	0.55	1, 128	0.00	0.80	1, 128	0.01
Age group × condition	0.34	1, 128	0.00	3.48	1, 128	0.03	0.80	1, 128	0.01
Study × age group × condition	9.90**	1, 128	0.07	0.00	1, 128	0.00	0.00	1, 128	0.00

*Note:* $\eta_{G}^{2}$ indicates generalized eta‐squared.

^*^ *p* < 0.05.

^**^ *p* < 0.01.

**TABLE A2:** Results of pairwise comparisons following up the significant study × age group × condition for timing of study appointments as dependent variable.

Age group	Condition	Comparison live versus video study
15 months	Nap	*M*_Diff_ = −15, *p* = 0.621
	No‐nap	*M*_Diff_ = −81, *p* = 0.008
24 months	Nap	*M*_Diff_ = −31, *p* = 0.296
	No‐nap	*M*_Diff_ = 92.6, *p* = 0.003
